# Characterization of Fungal FAD-Dependent AA3_2 Glucose Oxidoreductases from Hitherto Unexplored Phylogenetic Clades

**DOI:** 10.3390/jof7100873

**Published:** 2021-10-17

**Authors:** Sudarma Dita Wijayanti, Leander Sützl, Adèle Duval, Dietmar Haltrich

**Affiliations:** 1 Laboratory of Food Biotechnology, Department of Food Science and Technology, BOKU-University of Natural Resources and Life Sciences Vienna, Muthgasse 11, A-1190 Wien, Austria; sudarma.wijayanti@boku.ac.at (S.D.W.); leander.suetzl@boku.ac.at (L.S.); adele.duval@savencia.com (A.D.); 2 Department of Agricultural Product Technology, Brawijaya University, Veteran, 65145 Malang, East Java, Indonesia

**Keywords:** glucose dehydrogenase, glucose oxidase, GMC oxidoreductase, CAZy AA3_2, substrate specificity

## Abstract

The CAZy auxiliary activity family 3 (AA3) comprises FAD-dependent enzymes belonging to the superfamily of glucose-methanol-choline (GMC) oxidoreductases. Glucose oxidase (GOx; EC 1.1.3.4) and glucose dehydrogenase (GDH; EC 1.1.5.9) are part of subfamily AA3_2 and catalyze the oxidation of β-D-glucose at its anomeric carbon to D-glucono-1,5-lactone. Recent phylogenetic analysis showed that AA3_2 glucose oxidoreductases can be grouped into four major clades, GOx I and GDH I–III, and in minor clades such as GOx II or distinct subclades. This wide sequence space of AA3_2 glucose oxidoreductases has, however, not been studied in detail, with mainly members of GOx I and GDH I studied biochemically or structurally. Here, we report the biochemical characterization of four fungal glucose oxidoreductases from distinct, hitherto unexplored clades or subclades. The enzyme from *Aureobasidium subglaciale*, belonging to the minor GOx II clade, showed a typical preference for oxygen and glucose, confirming the correct annotation of this clade. The other three enzymes exhibited strict dehydrogenase activity with different substrate specificities. GDH II from *Trichoderma virens* showed an almost six-fold higher catalytic efficiency for maltose compared to glucose. The preferred substrate for the two GDH III enzymes from *Rhizoctonia solani* and *Ustilago maydis* was gentiobiose, a β(1→6) disaccharide, as judged from the catalytic efficiency. Overall, the newly studied AA3_2 glucose oxidoreductases showed a much broader substrate spectrum than the archetypal GOx from *Aspergillus niger*, which belongs to clade GOx I.

## 1. Introduction

The Carbohydrate-Active enZymes database classifies a number of enzymes involved in the transformation of carbohydrates based on their functional amino acid sequences, reaction mechanisms, and structural similarities (CAZy database; http://www.cazy.org/; accessed on 27 August 2021) [1]. It comprises glycoside hydrolases, carbohydrate esterases, polysaccharide lyases, glycosyltransferases, carbohydrate-binding modules, and enzymes with auxiliary activities (AA); the latter group includes various redox enzymes that act in conjunction with other CAZymes. Currently, the AA group is divided into 17 families, and some of these families (AA1, AA3, and AA5) are further divided into subfamilies [2]. The members of CAZy auxiliary activity family 3 (AA3) are FAD (flavin adenine dinucleotide)-dependent enzymes, belonging to the superfamily of glucose-methanol-choline (GMC) oxidoreductases. AA3 enzymes, and predominantly those of fungal origin, were shown to possess significant applied potential, e.g., in biomass utilization, in biosensor applications, or in the food industry [3]. Four AA3 subfamilies have been described based on their phylogenetic relatedness and substrate preferences [4]. Subfamily AA3_2 is a large and diverse group, including several phylogenetically distinct clades of genes that encode glucose oxidase (GOx; EC 1.1.3.4), glucose dehydrogenase (GDH; EC 1.1.5.9), aryl-alcohol oxidoreductases (AAOx; EC 1.1.3.7, and AADH; EC 1.1.1.90), as well as pyranose dehydrogenase (PDH; EC 1.1.99.29). Several of these clades of AA3_2 enzymes have not been studies in any detail, and possible functions of their member enzymes are only annotated because of similarities analyzed, for example, by sequence similarity networks [5].

GOx and GDH both catalyze the oxidation of β-D-glucose at its anomeric carbon to D-glucono-1,5-lactone. Where the oxidases efficiently utilize oxygen as an electron acceptor in their oxidative half-reaction, dehydrogenases show negligible reactivity with oxygen and employ alternative electron acceptors, including a variety of aromatic compounds or metal ions. In addition, some representatives of the AA3_2 glucose oxidoreductases only oxidize glucose and strictly discriminate against other sugars, for example, the archetypal glucose oxidase from *Aspergillus niger*, whereas other members of this group show much broader sugar substrate reactivities, oxidizing different mono- and even oligosaccharides. A good example for this latter type of enzymes is the glucose dehydrogenase from *Pycnoporus cinnabarinus*. Recently, it was shown that this enzyme shows much higher activity against laminaribiose (glucosyl-β(1→3)-glucose) than for glucose; in fact, it was suggested that this enzyme should be named as an oligosaccharide dehydrogenase [6].

**Figure 1:**
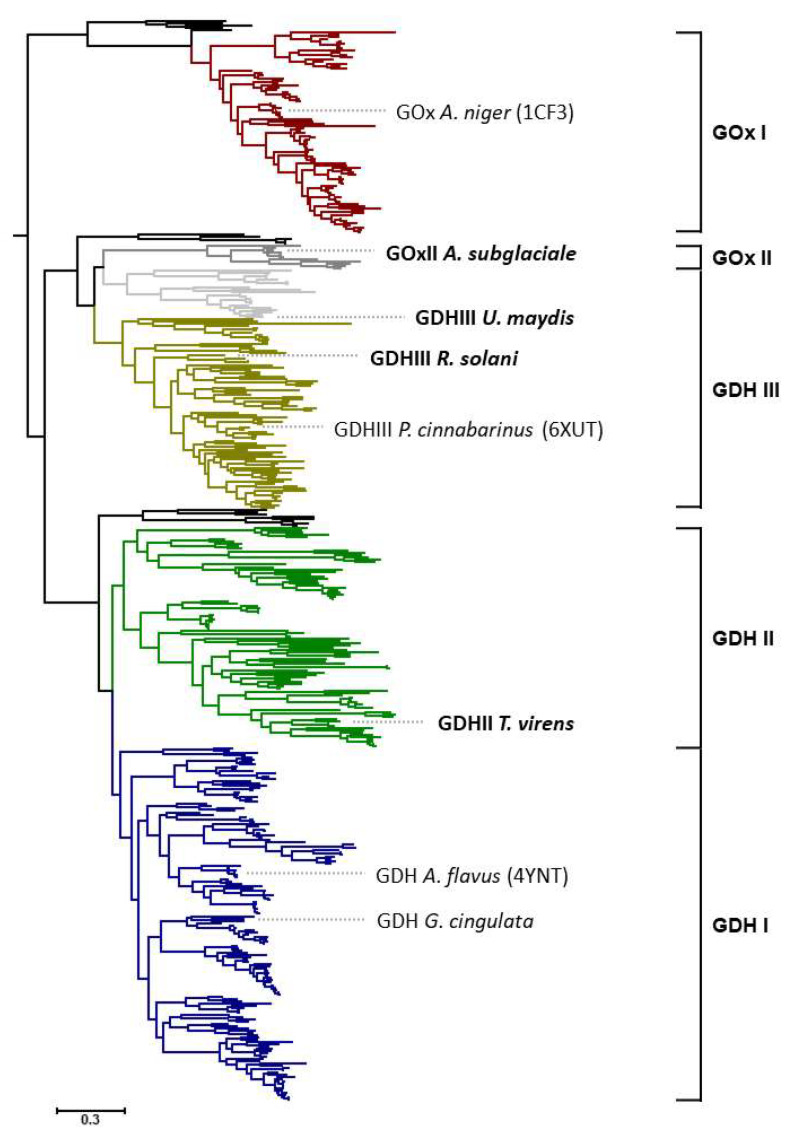
Phylogenetic tree of the AA3_2 glucose oxidoreductases and their detailed classification as previously reported [5]. The tree shows the main clades GOx I (**red**), GDH I (**blue**), GDH II (**green**), and GDH III (**yellow**). The minor GOx II clade is shown in dark grey, the minor subclade of GDH III containing only sequences of Ustilaginomycotina origin is shown in light grey. Novel GOx and GDH sequences selected for the present study are indicated in bold. The black bar indicates phylogenetic distance in amino acid substitution per site.

Sequences of AA3_2 glucose oxidoreductases were recently separated into four major clades, GOx I, GDH I, GDH II, and GDH III (Figure 1). The above-mentioned glucose dehydrogenase (oligosaccharide dehydrogenase) from *P. cinnabarinus* is positioned in the main clade of GDH III, and had been the only enzyme hitherto characterized from this clade [7]. Clades GDH I and GOx I (composed of Ascomycota sequences exclusively) are biochemically and structurally fairly well-characterized, with members such as *Aspergillus flavus* GDH [8,9], *Glomerella cingulata* GDH [10,11], or *A. niger* GOx, whereas the major clade GDH II (also composed of Ascomycota sequences exclusively) is completely unexplored. In addition to these well-defined clades, there are also some minor clades or subclades; however, these are only made up by a small number of available sequences [5]. One of these is a small subclade of GDH III, composed of 21 sequences of only Ustilaginomycotina origin, which, however, is distinguished from the main GDH III clade by an insertion of about 15 amino acids [5]. Another minor clade is GOx II, which is also only formed by Ascomycota sequences that closely associate with Basidiomycota sequences of clade GDH III, however. At the time of our previous study, only 11 sequences were included in this minor clade of narrow taxonomic distribution, limited to three orders of Pezizomycotina, i.e., Dothideales, Capnodiales, and Xylariales [5]. Based on the conservation of active-site residues that were shown to be important for reactivity of GOx I enzymes with oxygen (Thr110, Phe215, Phe351, Phe414 in *A. niger* GOx, 1CF3 [8,12]), we predicted that members of this minor clade should be oxidases, while other clades that were experimentally not characterized at all (GDH II) or only to a minor extent (GDH III) were predicted to be strict dehydrogenases based on these active-site residues. In a similar way, we also predicted the substrate specificity of the various glucose oxidoreductase clades by analyzing the conservation of active site residues attributed to glucose binding (Tyr68, Thr110, Asp424, Arg512, and Asn514 in *A. niger* GOx, 1CF3 [8,13,14]). We found that only clades GOx I and GDH I showed a high conservation of glucose-binding residues, while GOx II showed a reduced conservation, and GDH II and GDH III showed hardly any conservation of the analyzed residues. We therefore predicted that clades with less-conserved glucose-binding residues were more likely to also utilize other sugars as substrates.

It was the aim of our study to investigate the sequence space of AA3_2 glucose oxidoreductases in more detail, with emphasis on the currently unexplored clades and sub-clades. Since the selected sequences for this study show quite some phylogenetic distance and differences in their active site residues to the known and characterized enzymes, one can expect certain differences in the properties of these enzymes, for example pertaining to reactivity, substrate specificity, stability, etc.

In this work, we report the biochemical characterization of four fungal AA3_2 glucose oxidoreductases from distinct (sub)clades, which were heterologously expressed in *Komagataella phaffii* *(Pichia pastoris*). We selected a putative glucose oxidase sequence of *Aureobasidium subglaciale* (*As*GOxII) from the minor GOx II clade and three glucose dehydrogenase sequences from *Trichoderma virens* (*Tv*GDHII) from clade GDH II, *Rhizoctonia solani* (*Rs*GDHIII) from the main GDH III clade and from *Ustilago maydis* (*Um*GDHIII) belonging to the minor subclade of GDH III containing only sequences of Ustilaginomycotina origin. The positioning of these sequences in the phylogenetic tree together with the position of some well-characterized members of the AA3_2 glucose oxidoreductases is shown in Figure 1.

## 2. Materials and Methods

### 2.1. Strains, Media, and Chemicals

The *Komagataella phaffii* *(Pichia pastoris*) Mut^–^ strain was obtained from the Department of Biotechnology, BOKU University of Natural Resources and Life Sciences, Vienna, Austria. Deletion of *aox1* and *aox2* resulted in the Mut^–^ phenotype [15], and this strain was the host for recombinant expression of all genes used in this study. Electrocompetent *E. coli* strain NEB 5-alpha (New England Biolabs, Ipswich, MA, USA) was used for propagation of plasmids. *E. coli* cells were cultivated in a low-salt Luria-Bertani (LB) medium containing 10 g/L of peptone from casein, 5 g/L yeast extract, 5 g/L NaCl, and 25 mg/L of zeocin. YPD media containing 20 g/L of peptone from casein, 10 g/L yeast extract, 4 g/L glucose, 100 mg/L zeocin, and 15 g/L agar were used to grow *K. phaffii* transformants. Buffered methanol complex (BMMY) medium containing 20 g/L peptone from casein, 10 g/L yeast extract, 100 mM potassium phosphate buffer at pH 6.0, 13.4 g/L Yeast Nitrogen Base 10×, and 0.4 mg/L biotin 500× was used for gene expression in *K. phaffii*. All monosaccharides and disaccharides were purchased from Sigma-Aldrich (St. Louis, MO, USA), except for laminaribiose, which was purchased from Megazyme (Wicklow, Ireland), gentiobiose was from Carl Roth (Karlsruhe, Germany), and sophorose was from Serva (Heidelberg, Germany).

### 2.2. Genes and Protein Sequences Analysis

Four genes coding for AA3_2 glucose oxidoreductases from different subclades (Table S1) were selected for detailed studies: *A. subglaciale* glucose oxidase (*As*GOxII, GenBank: KEQ90431) from the minor GOx II clade, *T. virens* glucose dehydrogenase (*Tv*GDHII, GenBank: EHK19553) from the GDH II clade, *R. solani* (*Rs*GDHIII, GenBank: CEL62789), and *U. maydis* glucose dehydrogenase (*Um*GDHIII, GenBank: KIS68464) from the GDH III clade as indicated in Figure 1. All sequences were codon-optimized for expression in *K. phaffii* and ordered from BioCat GmbH (Heidelberg, Germany) cloned into the expression vector pPICZ A, using the restriction sites *Bst*BI and *Psp*OMI, resulting in a C-terminal tag for the expressed proteins consisting of a linker, a Myc-tag, a linker, and a (His)_6_-tag (-GGP-EQKLISEEDL-NSAVD-HHHHHH) in addition to the native sequence. Two genes of well-characterized members of the AA3_2 glucose oxidoreductases, *A. niger* glucose oxidase (*An*GOx) [16] and GDH I from *G. cingulata* (*Gc*GDH) [10,11,17,18] were also added to this study as references. Potential N-glycosylation sites of the mature proteins were predicted by NetNGlyc 1.0 (http://www.cbs.dtu.dk/services/NetNGlyc; accessed on 20 July 2021). Molecular masses, pI values, and possible signal sequences were calculated by using the programs Compute pI/Mw and SignalP hosted on the Expasy Proteomics server (http://www.expasy.ch; accessed on 20 July 2021).

### 2.3. Gene Expression and Production of Recombinant AA3_2 Glucose Oxidoreductases

The expression plasmids were linearized with *Bsi*WI and transformed into electrocompetent *K. phaffii* Mut^–^ cells. Transformants were selected on YPD zeocin plates, and three positive colonies each were selected for expression studies in shaken baffled flasks.

*K. phaffii* cells were cultivated on YPD plates containing 100 mg/L zeocin at 30 °C for 48 h and used as inoculum for pre-cultures in 20 mL liquid YPD medium containing 100 mg/L zeocin (20 h, 30 °C, 140 rpm). These pre-cultures were centrifuged (5 min, 3000× *g*, room temperature), the supernatant was decanted, and the cell pellet was resuspended and inoculated into 1 L baffled flasks containing 200 mL of BMMY medium to an OD_600_ of 1. Cultivations were performed with simultaneous feeds of 15 mL sorbitol 1 M as carbon source, and methanol at 2% of the final volume. Expression was performed at 30 °C, 140 rpm. Samples of the culture were taken regularly for the determination of cell density (OD_600_), extracellular protein concentration, and oxidase/dehydrogenase activity. After 72 h of cultivation, the cultures were harvested by centrifugation at 10,000× *g* for 15 min at 4 °C, and the cells were discarded. The empty pPICZ A vector, not containing any recombinant gene, was used as a negative control.

### 2.4. Enzyme Purification

The culture supernatant was loaded onto IMAC Ni-charged resin (5 mL HisTrap^TM^ HP column; GE Healthcare, IL, USA) followed by washing with buffer A (50 mM phosphate buffer, 50 mM NaCl, 20 mM imidazole pH 6.5) to remove unbound protein. The bound target enzyme was eluted with a linear gradient of 10 column volumes of buffer B (50 mM phosphate buffer, 50 mM NaCl, 500 mM imidazole pH 6.5). All buffers were filtered and degassed prior to use. The active, pooled fractions (confirmed by the O_2_/ABTS and/or DCIP standard assays) were concentrated, and the buffer was exchanged using an Amicon Ultra Centrifugal Filter Device (Merck Millipore, Massachusetts, USA) of 50 kDa cut-off via centrifugation at 4000 rpm, 4 °C for 30 min, to 50 mM phosphate buffer pH 6.5. The collected enzymes were filter-sterilized, aliquoted, and stored at –30 °C for further analysis.

### 2.5. Analysis of Molecular Properties

SDS-PAGE analysis was performed using Mini-PROTEAN TGX stain-free precast gels with a denaturing gradient of 4–15% (BioRad, Hercules, CA, USA). Precision Plus Protein Unstained standards were used as ladder for molecular mass identification. All procedures were carried out according to the manufacturer’s recommendation. Deglycosylation was conducted by treating the protein samples with PNGase F (New England Biolabs) under denaturing conditions as recommended by the manufacturer. Following SDS-PAGE, the proteins were electroblotted onto 0.2 µm nitrocellulose membranes (BioRad) using the Trans-Blot Turbo Transfer System (BioRad) according to the manufacturer’s instruction. The blot was then incubated in tris-borate saline (TBS) blocking solution (10 mM Tris-HCl pH 7.4, 2.5% *w/v* bovine serum albumin (BSA), and 150 mM NaCl) overnight at 4 °C, briefly rinsed with TBS-Tween (10 mM Tris-HCl pH 7.4, 150 mM NaCl, 0.05% Tween 20), and incubated with TBS-Tween buffer containing 0.5% BSA and penta-His Tag monoclonal antibody serum at 1/5000 dilution as primary antibody (Qiagen, Germantown, MD, USA) overnight at 4 °C. The membrane was washed three times using TBS-Tween buffer and then incubated with the secondary antibody, polyclonal rabbit anti-mouse immunoglobulins/HRP (Dako, Glostrup, Denmark) at a 1/2000 dilution, precision protein StrepTactin-HRP conjugate (BioRad) 1/10,000 dilution in TBS-Tween buffer, 0.5% BSA, for 1 h at room temperature. Finally, the Clarity western ECL reagent kit (BioRad) was used as recommended by the manufacturer to visualize protein bands.

UV-Vis spectra of purified enzymes in 50 mM potassium phosphate buffer (pH 6.5) were recorded from 250–550 nm at room temperature in both their oxidized and reduced states using a diode array-based UV-Vis spectrophotometer (Agilent 8453, Santa Clara, CA, USA), before and shortly after the addition of 100 mM D-glucose to the cuvette. A quartz cuvette (3 mm path length) was used for the measurements. The molar absorption coefficients of the respective enzymes at the wavelength maximum of around 450 nm were determined by precipitation of the polypeptide with trichloroacetic acid according to [19] and the molar absorption coefficient for released free FAD (Ɛ_450_ = 11.3 mM^−1^ cm^−1^).

High-Pressure Liquid Size Exclusion Chromatography coupled to multiangle light scattering (HPLC-SECMALS) was carried out to check the homogeneity and oligomeric state of the purified enzymes. The HPLC-LC20 system (Shimadzu Prominence LC20; Shimadzu Europe, Duisburg, Germany) with the refractive index detector RIF-10A, the photodiode array detector SPD-M20A (Shimadzu), and MALS Heleos Dawn8^+^ with QELY detector (Wyatt Technology, Santa Barbara, CA, USA) was used for the analysis. Superdex 200 10/300 GL (GE Healthcare) was equilibrated with phosphate-buffered saline (PBS) pH 7.4 containing 200 mM NaCl as the running buffer. Experiments were performed at a flow rate of 0.75 mL min^−1^ and 25 °C, and the resulting data were analyzed using the ASTRA 6 software (Wyatt Technology). Proper performance of the molar mass calculation was verified by analysis of a BSA sample as reference. Routinely, 80 μg of protein (in 5−100 μL running buffer) was loaded per run.

### 2.6. Characterization of AA_3 Glucose Oxidoreductases

#### 2.6.1. Enzyme Activity Assays and Protein Measurement

The concentration of protein was determined by Bradford’s assay using the Bio-Rad Protein assay kit containing BSA as standard, measured in a DU 800 UV/Visible Spectrophotometer (Beckman Coulter, CA, USA). Enzyme activity assays were performed spectrophotometrically in the EnSpire 2300 Multilabel Reader (Perkin Elmer, IL, USA) using 96-well plates. The standard assay for oxidase activity was a peroxidase-coupled test based on 10 mM 2,2’-azino-bis(3-ethylbenzothiazoline-6-sulphonic acid) (ABTS, ε_420_ = 36 mM^−1^ cm^−1^) and horseradish peroxidase at 47 U mL^−1^ at 420 nm [20], while dehydrogenase activity was determined with 0.3 mM 2,6-dichlorophenolindophenol (DCIP, ε_520_ = 6.9 mM^−1^ cm^−1^), 0.5 mM 1,4-benzoquinone (1,4-BQ, ε_290_ = 2.24 mM^−1^ cm^−1^), or 1 mM ferrocenium hexafluorophosphate (FcPF_6_, ε_300_ = 4.3 mM^−1^ cm^−1^) as electron acceptors. Sugar substrates were added as indicated. Unless otherwise stated, 100 mM glucose was used routinely as the electron donor substrate at pH 6.5. One unit of enzymatic activity was defined as the amount of enzyme that oxidizes 1 µmol of sugar per min under the assay conditions. The reaction stoichiometry of the electron donor glucose is 1 to the two-electron acceptors DCIP and 1,4-BQ but is 2 for the one-electron acceptor FcPF_6_. The reaction was followed for 180 s at 30 °C.

#### 2.6.2. Effect of pH and Temperature on Enzyme Activities

Optimal pH values of the enzyme activities were determined using potassium citrate buffer (pH 3–6), potassium phosphate buffer (pH 6–8), and tris-HCl buffer (pH 8–10), at 30 °C. All other conditions were as under standard assay conditions, and each measurement was taken in triplicate. The thermostability of GOx/GDHs was investigated both by thermal unfolding (melting temperature, T_m_) and thermal inactivation (T_50_). In order to monitor protein unfolding, the ThermoFAD assay [21] was used for the determination of the T_m_ value, the melting temperature midpoint of the transition. Each enzyme sample was diluted to a final concentration of 1 mg/mL using a buffer with a pH corresponding to the optimum of activity and measured in triplicates with 25 μL aliquots in each well. A single-color real-time PCR cycler (i-Cycler, BioRad), providing a MyiQ Optics Module and SYBR-Green filters (523–543 nm), was used to record the signals during the heat treatment. All the samples were heated in 0.5 °C steps (20 s per step) from 30° to 95 °C using the thermal gradient program of the PCR machine. The fluorescence signal was measured at the end of each heating step.

Thermal inactivation (T_50_), defined as the temperature at which 50% of the original activity is retained after a 20 min heating step, was investigated by incubating enzyme samples at the respective optimum pH of activity at different temperatures ranging from 30 to 65 °C for 20 min using the gradient temperature module of the C1000 Thermal Cycler (BioRad). The reaction was then stopped by immediately cooling the treated samples on ice, and the residual activity was measured in triplicates under standard condition against 0.5 mM 1,4-BQ as electron acceptor and 100 mM glucose as substrate.

#### 2.6.3. Reactivity with Sugar Substrates

The specific activities of purified enzyme samples were determined for different mono- and disaccharides: D-glucose, methyl-α-D-glucopyranoside, D-xylose, D-galactose, D-arabinose, D-mannose, D-fructose, trehalose, sucrose, maltose, isomaltose, lactose, cellobiose, gentiobiose, laminaribiose, and sophorose. The measurements were taken under standard assay condition against 0.5 mM 1,4-benzoquinone as electron acceptor and 100 mM of the sugar substrate as previously described in [11].

#### 2.6.4. Steady-State Kinetic Measurements

Apparent steady-state kinetic constants for different electron donor substrates at varied concentrations were determined using 1,4-BQ as electron acceptor at a fixed concentration of 0.5 mM. D-glucose, D-xylose, D-galactose, D-mannose, maltose, isomaltose, lactose, laminaribiose, and gentiobiose in the range of 1–500 mM were used as indicated. Catalytic constants were measured at pH 6.5, 30 °C and calculated using nonlinear least-square regression by fitting the observed data to the Michaelis–Menten equation, v = v_max_ [S]/K_m_^+^[S] (Sigma Plot 12, Systat, Chicago, IL, USA). All measurements were conducted in triplicates, and the results are given as mean value ± standard deviation (SD) with *p* < 0.05.

### 2.7. Structure and Model Analysis

The sequence alignment and sequence identity matrix were created by using the alignment tool ClustalO (https://www.ebi.ac.uk/Tools/msa/clustalo/; accessed on 20 July 2021) [22]. The SWISS-MODEL (Swiss Institute of Bioinformatics, Lausanne, Switzerland) protein homology-modeling server [23] was used to create a three dimensional model of the four AA3_2 glucose oxidoreductases, and the Protein Homology/analogy Recognition Engine V 2.0 (Phyre^2^) was chosen to select the best homology models of the glucose-ligated active sites and suitable templates [24]. All models were analyzed and visualized using PyMol 2.4 (http://pymol.org/; accessed on 14 June 2021).

## 3. Results

### 3.1. Expression and Purification of Recombinant AA3_2 Glucose Oxidoreductases

The detailed phylogenetic tree of the AA3_2 glucose oxidoreductases indicates that previous biochemical studies focused on members of clades GOx I and GDH I, while the other clades are essentially unexplored except for a fungal glucose dehydrogenase of clade GDH III from *P. cinnabarinus*. In order to test whether the sequences within unexplored clades and subclades indeed code for active glucose oxidoreductases, we randomly picked eight sequences covering a wide sequence space in the unknown parts of the GOx/GDH tree (EKD04919, *Trichosporon asahii* var. *asahii*; EHA25730, *Aspergillus niger*; KEQ90431, *Aureobasidium subglaciale*; KIS68464, *Ustilago maydis*; CEL62789, *Rhizoctonia solani*; KXT14014, *Pseudocercospora musae*; KEQ88331, *Aureobasidium pullulans*; and EHK19553, *Trichoderma virens*, given here with their GenBank identifiers) and expressed the corresponding genes in *K. phaffii*. As indicated by SDS-PAGE analysis (data not shown), four out of these eight preliminary expression experiments yielded proteins of the correct size (molecular masses between 61 and 71 kDa) and showed activity (GOx or GDH activity) according to the proposed functional annotation of the various subclades.

These four successfully expressed fungal members of AA3_2 glucose oxidoreductases were selected for further biochemical characterization: GOx from *A. subglaciale* (*As*GOxII) from the GOx II clade, GDH from *T. virens* (*Tv*GDHII) from clade GDH II, GDH from *R. solani* (*Rs*GDHIII), and *U. maydis* (*Um*GDHIII) from clade GDH III.

SDS-PAGE analysis followed by Western blot-immunostaining against the His-Tag were carried out to check whether these four genes were successfully expressed. Positive results for the four selected proteins when using anti-His antibodies are shown in Figure S1A. Further, activity assays were conducted to select the best producing clones giving highest activity. No oxidase/dehydrogenase activity was observed in *K. phaffii* cells harboring the empty vector (without an inserted gene). Culture supernatants of selected *K. phaffii* clones were used for the purification. Purification of recombinant AA3_2 glucose oxidoreductases was 1.2 to 13-fold with 60–85% of recovery, yielding approximately 8 to 110 mg of purified recombinant AA3_2 glucose oxidoreductases from 200 mL of culture broth.

The molecular mass of recombinant AA3_2 glucose oxidoreductases was investigated by SDS-PAGE (Figure S1B) and SEC-MALS analysis (Figure S2). SDS-PAGE of *As*GOxII, *Tv*GDHII, and *Rs*GDHIII showed broad and diffuse bands indicating glycosylation as also suggested by several potential glycosylation sites (Table S1). The molecular mass of native, glycosylated recombinant *As*GOxII, *Tv*GDHII, and *Rs*GDHIII were found to be around 192, 97 and 103 kDa, respectively, by SEC-MALS. After deglycosylation under denaturing conditions using PNGase F, a single, sharp band of smaller mass was obtained in SDS-PAGE for these three enzymes. The molecular masses of *As*GOxII, *Tv*GDHII, and *Rs*GDHIII were estimated by SDS-PAGE and comparison to standard proteins to be 68.1, 65, and 66.9 kDa, respectively. These values correspond well to the theoretical calculated molecular mass for a single mature subunit including the C-terminal (His)_6_-tag (Table S1). These data also suggest that native, glycosylated *As*GOxII forms a dimer, where both *Tv*GDHII and *Rs*GDHIII are monomeric proteins. A comparison of calculated molecular masses with masses resulting from SEC-MALS analysis suggests a glycosylation of 29%, 33%, and 35% for *As*GOxII, *Tv*GDHII, and *Rs*GDHIII, respectively.

Western blot-immunostaining of the *Um*GDHIII-containing supernatant gave a strong band of around 40–50 kDa (Figure S1A), which is significantly smaller than expected. SDS-PAGE of the purified enzyme showed several bands for the glycosylated form, and two clear bands of 19 and 47 kDa after deglycosylation. In contrast, SEC-MALS analysis of native, glycosylated *Um*GDHIII gave one single, sharp peak, indicating a homogenous enzyme preparation and a molecular mass of 79 kDa. In order to analyze the nature of the two bands from SDS-PAGE, we used mass spectrometry, which confirmed that both bands correspond to the *Um*GDHIII sequence, the 19 kDa polypeptide being the N-terminal and the 47 kDa polypeptide the C-terminal fragment of the intact sequence. We assume that this cleavage occurred during sample preparation for SDS-PAGE since SEC-MALS showed a homogenous protein preparation and the untreated, purified enzyme showed significant activity. *Um*GDHIII was estimated to have 16% glycosylation based on the comparison of calculated molecular mass with mass measured by SEC-MALS.

All purified glucose oxidoreductase preparations showed a distinct, bright yellow color and displayed a UV-Vis spectrum that is typical for flavin-containing proteins with absorption maxima at around 390 and 450 nm (Figure S3). These peaks disappeared after addition of 100 mM D-glucose and the reduced enzymes became colorless. Trichloroacetic acid treatment released the flavin from the precipitated polypeptide into the supernatant, indicating that the glucose oxidoreductases studied here contain a non-covalently bound flavin, presumably FAD in accordance with other glucose oxidoreductases of the GMC superfamily.

### 3.2. Biochemical Properties of AA3_2 Glucose Oxidoreductases

#### 3.2.1. Catalytic Properties Using Different Electron Acceptors

Screening for catalytic activities was conducted using D-glucose as the electron donor substrate and four different electron acceptors, oxygen (air saturation) together with the peroxidase-coupled ABTS assay measuring the formed hydrogen peroxide for monitoring oxidase activity, 2,6-dichlorophenol-indophenol (DCIP) as commonly used substrate for the dehydrogenase activity assay, and in addition the alternative electron acceptors 1,4-benzoquinone (1,4-BQ) and the ferrocenium ion (ferrocenium hexafluorophosphate, FcPF_6_). As summarized in Table 1, only *As*GOxII showed significant activity with oxygen as electron acceptor while *Tv*GDHII, *Rs*GDHIII, and *Um*GDHIII gave no measurable oxidase activity. *An*GOx was used as a reference oxidase and showed considerably higher specific activity than *As*GOxII. All the glucose oxidoreductases tested were able to reduce the various alternative electron acceptors employed, albeit to a varying extent, and typically highest specific activities were measured with 1,4-BQ. The reference dehydrogenase GDH from *Glomerella cingulata* (*Gc*GDH) again showed considerably higher activities than the dehydrogenases from the other clades. These results proved that *As*GOxII is an oxidase whereas *Tv*GDHII, *Rs*GDHIII, and *Um*GDHIII are true dehydrogenases as proposed by the functional classification of the phylogenetic tree [5].

#### 3.2.2. pH Dependence of AA3_2 Glucose Oxidoreductase Activities

**Figure 2:**
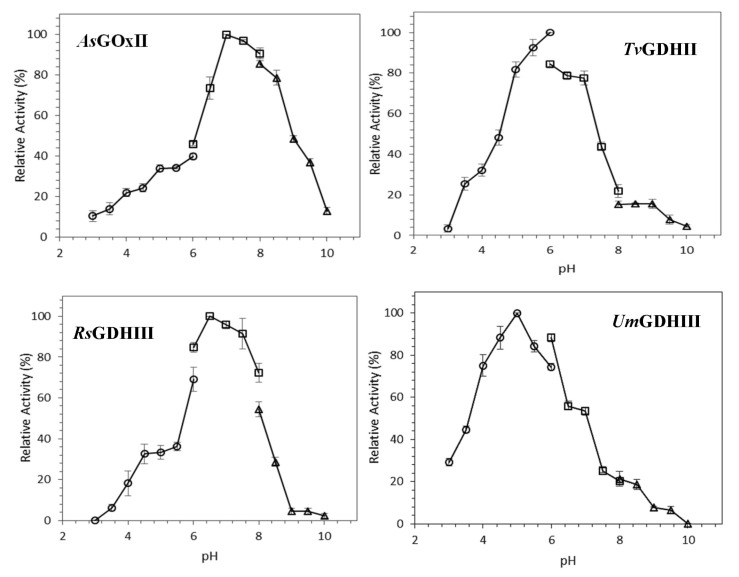
Influence of the pH value on the activity of AA3_2 glucose oxidoreductases. Relative activities measured at various pH values for the substrate couple D-glucose/1,4-BQ. The buffers used were 50 mM potassium citrate (o), potassium phosphate (Δ), and Tris-HCl (Δ). Values represent the mean ± SD of triplicate independent measurements.

The pH dependence of GOx/GDH activities was determined using glucose and 1,4-BQ as substrates (Figure 2). Highest activities are observed at pH 7 for *As*GOxII, pH 6 for *Tv*GDHII, pH 6.5 for *Rs*GDHIII, and pH 5.0 for *Um*GDHIII. All GOx/GDHs activities are relatively low at pH values below 4. About 75% of maximum activity was found at a pH range of 6.5–8.5, 5.0–7.0, 6.0–7.5, and 4.0–6.0 for *As*GOxII, *Tv*GDHII, *Rs*GDHIII, and *Um*GDHIII, respectively.

#### 3.2.3. Effect of Temperature on AA3_2 Glucose Oxidoreductase Stability

The effect of temperature on the stability of GOx/GDHs was compared (Figure S4). Thermal unfolding experiments using the ThermoFAD method showed that *As*GOxII had the lowest thermal unfolding transition value (T_m_) of 48 °C, while the other enzymes showed T_m_ values ranging from 52 to 56 °C (Table 2). Based on the thermostability index, the temperature at which 50% of the activity is lost after a 20-min incubation (T_50_), *Um*GDHIII showed the highest thermostability in accordance with the unfolding studies. The reference enzyme *An*GOx showed significantly higher thermostability than the novel enzymes studied, while *Gc*GDH showed lower thermostability than most other enzymes.

### 3.3. Reactivity with Different Sugar Substrates

**Figure 3:**
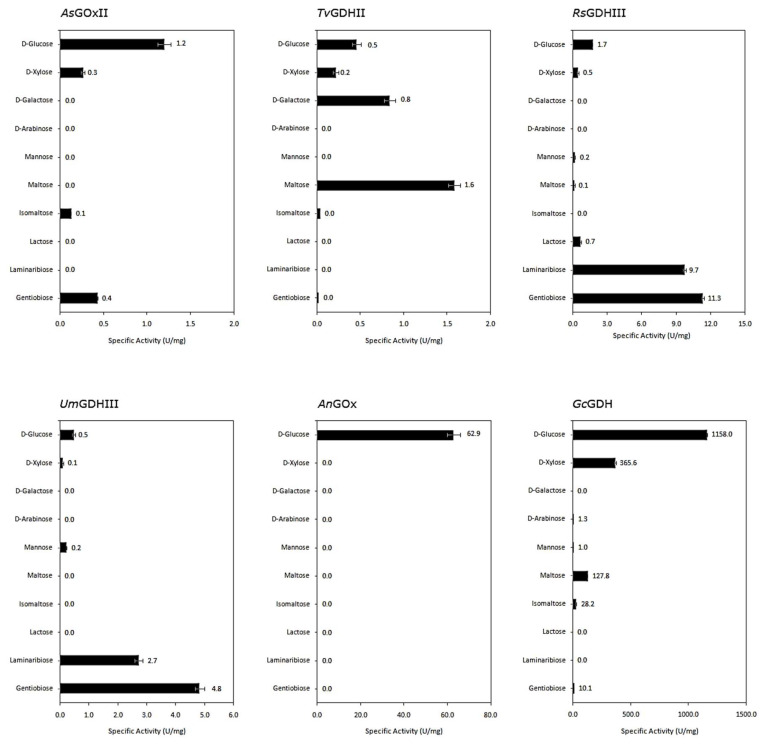
Sugar substrate spectrum of AA3-2 glucose oxidoreductases. Activities were measured using 0.5 mM 1,4-benzoquinone as electron acceptors and 100 mM of sugar substrates. Values represent the mean ± SD of triplicate independent measurements.

Various monosaccharides and glucose-containing disaccharides of different glycosidic linkage, each at a concentration of 100 mM, were tested as possible electron donors to compare the spectrum of sugar substrates oxidized by the different AA3_2 enzymes (Figure 3).

In general, our novel AA3_2 glucose oxidoreductases showed lower specific activities with the various sugars in comparison to the well-known *An*GOx and *Gc*GDH, but they show a more diverse substrate preference. Substitution of the hydroxyl group at C-1 as in methyl-D-glucopyranoside resulted in a complete loss of activity, indicating that the enzymes oxidize sugar substrates exclusively at the C-1 position. Furthermore, all glucose oxidoreductases showed no activity toward fructose, sucrose, trehalose, and the β(1→2) disaccharide, sophorose. Among the newly studied enzymes, only *As*GOxII showed a preference for D-glucose, with lower activity toward gentiobiose, xylose, and isomaltose. In contrast, the other three enzymes showed different specificity patterns toward sugar substrates, and glucose was not their preferred substrate. *Tv*GDHII preferentially oxidized maltose, followed by galactose, the C-4 epimer of glucose, whereas the specific activity for glucose was two-fold lower. Both GDHs from clade III, *Rs*GDHIII and *Um*GDHIII, exhibited highest reactivity in the presence of gentiobiose, glucosyl-β(1→6)-glucose, and also showed good activity toward laminaribiose, glucosyl-β(1→3)-glucose. *Rs*GDHIII showed the broadest sugar substrate spectrum oxidizing lactose, xylose, mannose, maltose, and cellobiose as well.

### 3.4. Kinetic Properties of AA3_2 Glucose Oxidoreductases

The apparent steady-state kinetic constants were determined for the different enzymes and their preferred sugar substrates with 1,4-BQ kept at a constant concentration, with results summarized in Table 3. In general, the catalytic efficiencies k_cat_/K_m_ measured for glucose and the four selected enzymes are significantly lower than for the two reference enzymes, *An*GOx and *Gc*GDH. Based on these catalytic efficiencies, only *As*GOxII preferred glucose as its electron donor substrate, whereas the other three enzymes showed clear preferences for different disaccharides over glucose, maltose in the case of *Tv*GDHII (59.2 vs. 10.8 M^−1^ s^−1^), and gentiobiose in the case of both *Rs*GDHIII (408 vs. 96.7 M^−1^ s^−1^) and *Um*GDHIII (122.3 vs. 18.4 M^−1^ s^−1^). These higher catalytic efficiencies are mainly a result of the increase in the catalytic constants k_cat_ measured for the disaccharides, whereas the Michaelis constants K_m_ are comparable for the preferred disaccharides and glucose.

### 3.5. Amino Acid Sequence Comparison

We performed a pair-wise sequence comparison between our four newly studied glucose oxidoreductases and structurally characterized members of the AA3_2 glucose oxidoreductases-*An*GOx (PDB entry 1CF3) of clade GOx I, a glucose dehydrogenase of clade GDH I from *Aspergillus flavus* (*Af*GDH; PDB entry 4YNT) as well as a glucose dehydrogenase of clade GDH III from the basidiomycete *P. cinnabarinus* (*Pc*GDHIII; PDB entry 6XUT). The structure of this latter enzyme was elucidated only recently, and because of the preferential activity of this enzyme toward oligosaccharides containing a β(1→3)-linked reducing glucose moiety such as laminaribiose or 1,3:1,4 β-glucotriose B (3^1^-β-D-cellobiosyl-glucose), it was termed oligosaccharide dehydrogenase [6]. For reasons of consistency with the nomenclature of the other AA3_2 members, we are here referring to this enzyme as *Pc*GDHIII. Both *Rs*GDHIII and *Um*GDHIII show high sequence similarity to *Pc*GDHIII (59.73 and 45.92%, respectively), which reflects their classification to the GDH III clade and the further phylogenetic distance of *Um*GDHIII to *Pc*GDHIII compared to *Rs*GDHIII (Figure 1). High sequence similarity was also found for *Af*GDH of clade GDH I and *Tv*GDHII of clade GDH II (42.20%). Not surprisingly, the sequence similarities of the new AA3_2 representatives and *An*GOx was relatively low, ranging from 31.42% to 34.54%, indicating the classification into separate clades of the phylogenetic tree (Table S2).

### 3.6. Structural Models of the Active Sites

**Figure 4:**
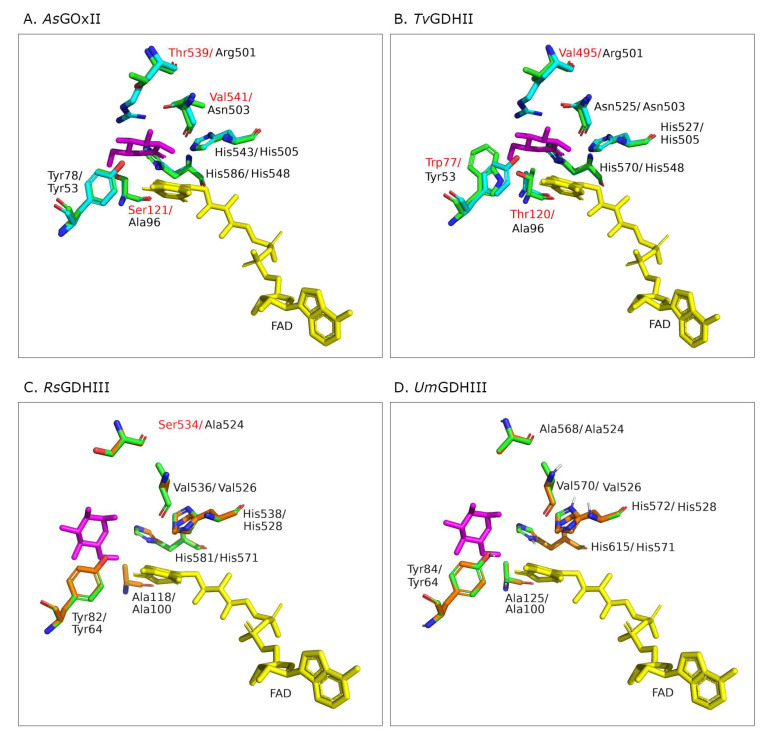
Superimposition of the structural models of AA3_2 glucose oxidoreductases (**green**) and the best model templates: *As*GOxII and *Tv*GDHII were superimposed to *Af*GDH (4YNU; **cyan**) based on the six residues responsible for glucose binding [8], while *Rs*GDHIII and *Um*GDHIII were superimposed to *Pc*GDHIII (6XUU; **orange**) [6]. Non-conserved active site residues in the respective AA3_2 glucose oxidoreductases are indicated in red. Glucose is shown in magenta.

The high specificity of *An*GOx toward β-D-glucose and its selectivity over D-xylose has been attributed to a highly specialized active-site architecture, characterized by conserved Tyr, Thr/Ser, Arg, and Asn residues and the catalytic His pair (Y68, T110, R512, N514, H516, and H559 in *An*GOx, 1CF3), which form hydrogen bonds to all five hydroxyl groups of β-D-glucose [8,16]. In order to compare glucose binding and its interaction with active-site residues of the AA3_2 glucose oxidoreductases, we prepared structural models of the active sites ligated with a glucose molecule. The best model templates proposed by Phyre^2^ were *Af*GDH (4YNT) for *As*GOxII as well as *Tv*GDHII, and *Pc*GDHIII (6XUU) for *Rs*GDHIII and *Um*GDHIII. The positions of the FAD and glucose in the homology models were taken from the structure of either *Af*GDH or *Pc*GDHIII (Figure 4).

## 4. Discussion

In a recent detailed analysis of enzymes of the GMC superfamily, we used sequence similarity networks to cluster large numbers of fungal GMC sequences and to annotate them according to their functionality. These functional clusters were then further analyzed with regards to their sequences and phylogeny [5]. One of the clusters of interest comprised FAD-containing AA3_2 glucose oxidoreductases, enzymes of significant applied potential. AA3_2 glucose oxidoreductases oxidize β-d-glucose at its anomeric carbon to yield D-glucono-1,5-lactone, while they can efficiently use oxygen as electron acceptor in the case of glucose oxidases, or oxygen can only be an ineffective electron acceptor in the case of glucose dehydrogenases. Based on the analysis of these sequences, we previously proposed four distinct major clades for the cluster containing the fungal AA3_2 glucose oxidoreductases [5]: Clade GOx I, which in the phylogenetic analysis is completely separated from the other clades and comprises glucose oxidases, GDH I, GDH II, and GDH III, which comprises glucose dehydrogenases, as well as several minor clades such as GOx II or subclades of GDH III. Up until now, several of these clades had functionally not been characterized at all, and others only had a single characterized representative. Although we predicted based on the conservation of certain active site residues that GOx II contains less glucose-specific oxidases, and GDH II as well as GDH III contain dehydrogenases with a wider substrate spectrum, experimental confirmation was still missing. We now report the biochemical characterization of AA3_2 glucose oxidoreductases of hitherto unstudied clades (such as GDH II or GOx II) or distinct branches of only poorly characterized clades (GDH III) and show that the predicted functional annotations of the AA3_2 glucose oxidoreductase phylogenetic tree hold true.

The AA3_2 glucose oxidoreductase of *A. subglaciale,* with its sequence belonging to clade GOx II, was shown to be indeed an oxidase as predicted even though it is phylogenetically closer to clade GDH III than to GOx I. Considering these results, we suggest that the close phylogenetic relationship between GOx I and GDH III does not necessarily portray the correct evolutionary history of the *gox* gene, but that GOx I and GOx II in fact evolved in parallel and are directly descending from a common ancestor. The close relation to GDH III could rather be an artifact of a generally high sequence identity within the group of AA3_2 glucose oxidoreductases and a low number of GOx II sequences, resulting in a lack of phylogenetically relevant positions in the alignment, which could lead to the misplacement of the GOx II branch within the tree. This seems to be very likely the case because of three reasons: (i) GOx I and GOx II are the only two clades in the AA3_2 glucose oxidoreductase tree, whose the genes show an unusual low number of exons (>90% of their sequences show either 1 or 2 exons, while GDH I, II, and III show 3.4, 3.0 and 10.0 exons, respectively); (ii) the occurrence of GOx I and GOx II genes in fungi does not overlap, which makes it likely that they are orthologs (both of these points are already discussed in [5]; and (iii) they are the only two clades harboring oxygen reactivity. Applying Occam’s razor, we think it more likely that the phylogenetic calculations are ambiguous. The other three AA3_2 glucose oxidoreductases that were studied, *Tv*GDHII, *Rs*GDHIII, and *Um*GDHIII, showed negligible activity with oxygen using the standard ABTS assay. They oxidized glucose in the present of suitable alternative electron acceptors though and were thus shown to be true dehydrogenases, also confirming their functional annotation from the phylogenetic tree of AA3_2 glucose oxidoreductases.

Based on the current results regarding the reactivity of *As*GOxII with xylose, the currently proposed role of a Ser or Thr residue as the major factor for discrimination against xylose might be questioned. The glucose oxidases from *A. niger* and *Penicillum amagasakiense* (*Pa*GOx), both belonging to clade GOx I, show high preference for glucose as their electron donor and efficiently discriminate against other monosaccharides such as xylose or mannose as judged from the catalytic efficiencies [25]. A serine or threonine residue forming a H-bond to 6-OH of glucose in members of GOx I (Ser114 in *Pa*GOx, Thr110 in *An*GOx) was proposed to be responsible for the efficient discrimination between glucose and xylose [14]: both *Af*GDH and *Gc*GDH are lacking this Ser or Thr residue but have an alanine residue at the corresponding position, and both show significant activity with xylose. *As*GOxII has a serine residue (Ser 121; Figure 4A) at the equivalent position; nevertheless, it oxidizes xylose comparably well. Even though its highest catalytic efficiency is with glucose, it showed a more relaxed substrate preference toward xylose and even disaccharides than the two GOx I enzymes *Pa*GOx and *An*GOx. The absence or presence of a H-bond to 6-OH in glucose therefore is not the only mechanism to discriminate between glucose and xylose by AA3_2 glucose oxidoreductases.

The active site of *Pc*GDHIII (6XUU; *Pc*ODH) was shown to be located at the bottom of a large funnel-shaped cavity. The catalytic His pair is located in the proximity of the isoalloxazine ring, and the side chains of these histidines are stabilized by H-bonds with a Gln and a Glu. Most of the other residues forming the active-site cavity of *Pc*GDHIII are of aromatic or hydrophobic nature [6]. Active-site residues around the proposed substrate-binding site of *Pc*GDHIII and *Rs*GDHIII as well as *Um*GDHIII are well-conserved as is the catalytic His pair (Figure 4C,D). The crystal structure of *Pc*GDHIII complexed with its two sugar substrates glucose and laminaribiose showed that sugar binding is not proceeding predominantly through H bonds but through CH–π interactions with aromatic residues. These aromatic residues are Phe421 (from the substrate-binding loop), Phe416, and Trp430 (*Pc*GDHIII 6XUU). Additionally, Tyr64 in *Pc*GDHIII stabilizes sugar binding through van der Waals interactions and a polar contact. These four residues are highly conserved in *Rs*GDHIII as well as *Um*GDHIII (only the position of Phe421 is substituted by a Tyr in *Rs*GDHIII), as they are typically also in other members of clade GDH III [6]. The funnel-shaped active site can accommodate two molecules of glucose, and it was proven that *Pc*GDHIII shows significantly higher catalytic efficiency for the disaccharide laminaribiose than for glucose, mainly because of a considerably more favorable Michaelis constant for the disaccharide. Both *Rs*GDHIII and *Um*GDHIII showed equally funnel-shaped active-site geometries in the model. Interestingly, the catalytic efficiency of both enzymes for laminaribiose is lower than that for glucose; however, both oxidize the disaccharide gentiobiose more efficiently than glucose, which is mainly the result of an increased catalytic constant compared to the reactivity with glucose.

The GDH from *T. virens* is the only fungal FAD-GDH studied from clade GDH II so far, and it is also the only AA3_2 glucose oxidoreductase reported to date that shows higher catalytic efficiency toward maltose compared to glucose. Several fungal AA3_2 glucose dehydrogenases were shown to accept maltose as a substrate; however, this activity is typically low to moderate [26,27,28].

All four newly studied enzymes generally showed a higher substrate diversity than is the case for previously studied glucose oxidoreductases of clades GOx I and GDH I. Only *As*GOxII showed a preference for glucose but was also accepting xylose, isomaltose, and gentiobiose. The other enzymes preferred maltose, in the case of *Tv*GDHII, and gentiobiose, in the case of *Rs*GDHIII and *Um*GDHIII, over glucose. These results are in good agreement with our predicted functional annotations of the hitherto uncharacterized clades that were based on the conservation of glucose-binding active site residues. Nevertheless, the coverage of the characterized sequence space, especially in the case of the GDH II clade, is still very low, and general statements about the different clades must be considered with care. It is striking though that two major clades of glucose oxidoreductases (GDH II and GDH III) are apparently comprising enzymes that are not primarily oxidizing glucose, and the name glucose oxidoreductase for this group of enzymes could be debated. Nevertheless, we are still referring to the name glucose oxidoreductases here, and did not adopt the term oligosaccharide dehydrogenases for the GDH III enzymes as had been suggested previously [6]. The reason for this is that all glucose oxidoreductases (GOx I-II and GDH I-III) share a close phylogenetic relation, and we want to avoid confusion with the CAZy auxiliary activity family 7, which is comprised of oligosaccharide oxidoreductases [29]. As was already discussed for other GMC oxidoreductases [4,6,11,30], the second, oxidative, half-reaction of these enzymes might indeed be the biological more relevant one, producing hydrogen peroxide or hydroquinones to fuel other lignocellulose-decomposing enzymes [11,31,32], reducing phenoxy radicals to prevent lignin (re)polymerization, or detoxify quinones that are secreted by plants as a defense mechanism against fungi [11]. The electron acceptor preference for 1,4-benzoquinone, which we observed for our newly characterized glucose oxidoreductases, might support this theory. Unfortunately, we are still lacking detailed information about the range of naturally occurring quinones and phenols that can be employed as electron acceptors by AA3_2 oxidoreductases to date.

## 5. Conclusions

In conclusion, we characterized four fungal AA3_2 glucose dehydrogenases from distinct clades with respect to their substrate specificities and biochemical properties. The corresponding sequences were taken from genome data and were previously only hypothetically annotated as glucose oxidases or dehydrogenases. We could show that they in fact oxidize glucose in the presences of oxygen or alternative electron acceptors in agreement to the positioning in the various clades or subclades. We also confirmed that GDHs from clade II and III showed highest activities toward disaccharides with different linkages, rather than toward glucose, which might also be of potential use for broader applications in the future, e.g., in specific enzyme-based biosensors or in biocatalytic oxidation of these oligosaccharides. For example, the glucose dehydrogenase from *T. virens* (*Tv*GDHII) with its pronounced activity for maltose could serve as a bioelement for the construction of a maltose sensor useful for the brewing industry.

## Figures and Tables

**Table 1:** Specific activities (U/mg) of various AA3_2 glucose oxidoreductases using D-glucose as electron donor substrate and various electron acceptors.

Enzymes	O_2_	DCIP	1,4-BQ	FcPF_6_
*As*GOxII	0.621 ± 0.017	0.0068 ± 0.0042	1.46 ± 0.03	0.0505 ± 0.0082
*Tv*GDHII	-	0.461 ± 0.012	0.423 ± 0.104	0.344 ± 0.011
*Rs*GDHIII	-	0.755 ± 0.054	2.95 ± 0.11	0.558 ± 0.113
*Um*GDHIII	-	0.0853 ± 0.0062	0.684 ± 0.082	0.218 ± 0.004
*An*GOx	79.5 ± 0.6	0.0108 ± 0.0113	60.6 ± 0.3	8.34 ± 0.51
*Gc*GDH	-	659 ± 74	1038 ± 40	1247 ± 52

Activities were measured at pH 6.5 and 30 °C using oxygen (air saturation), 3 mM DCIP, 1 mM FcPF_6_ or 0.5 mM 1,4-benzoquinone as electron acceptors in the oxidative half reaction, and 100 mM of D-glucose as electron donor. –no activity detected.

**Table 2:** Thermostability determination of GOx/GDHs.

Enzymes	T_m_ (°C)	T_50_ (°C)
*As*GOxII	48.4 ± 0.3	47.9 ± 0.4
*Tv*GDHII	52.9 ± 0.1	52.1 ± 0.3
*Rs*GDHIII	52.2 ± 0.4	51.5 ± 0.3
*Um*GDHIII	55.9 ± 0.4	53.2 ± 0.2
*An*GOx	63.8 ± 0.6	59.3 ± 0.2
*Gc*GDH	50.0 ± 0.2	50.0 ± 0.3

**Table 3:** Apparent steady-state kinetic constants of various AA3_2 glucose oxidoreductases. The concentration of the sugars was varied while using 1,4-benzoquinone as electron acceptor at a fixed concentration of 0.5 mM. Measurements were performed at pH 6.5 and 30 °C. Highest catalytic efficiencies are shown in bold.

Electron Donor		*As*GOxII	*Tv*GDHII	*Rs*GDHIII	*Um*GDHIII	*An*GOx	*Gc*GDH
Glucose	K_m_ (mM)	**21.3 ± 2.5**	55.7 ± 2.6	63.6 ± 1.3	12.5 ± 0.4	8.44 ± 1.46	11.2 ± 0.3
	k_cat_ (s^−1^)	**2.58 ± 0.59**	0.599 ± 0.055	6.15 ± 0.32	0.230 ± 0.009	47.4 ± 2.8	138.6 ± 1.5
	k_cat_/K_m_ (M^−1^ s^−1^)	**120.5**	10.8	96.7	18.4	5733	12,400
Xylose	K_m_ (mM) k_cat_ (s^−1^) k_cat_/K_m_ (M^−1^ s^−1^)	65 ± 12 1.78 ± 0.11 27.9	194 ± 14.2 0.431 ± 0.1 2.22	145 ± 15 2.29 ± 0.18 15.8	136.6 ± 7.4 0.161 ± 0.021 1.2	n.d.	21.0 ± 0.6 ^a^ 40 ± 1.5 ^a^ 1900 ^a^
Galactose	K_m_ (mM) k_cat_ (s^−1^) k_cat_/K_m_ (M^−1^ s^−1^)	n.d.	42.6 + 2.4 0.768 + 0.017 18	n.d.	n.d.	n.d.	n.d.
Mannose	K_m_ (mM) k_cat_ (s^−1^) k_cat_/K_m_ (M^−1^ s^−1^)	n.d.	n.d.	540 ± 15 0.591 ± 0.021 1.09	43.6 ± 8.4 0.267 ± 0.025 6.21	n.d.	n.d.
Maltose	K_m_ (mM) k_cat_ (s^−1^) k_cat_/K_m_ (M^−1^ s^−1^)	n.d.	**32.7 ± 1.5** **1.93 ± 0.05** **59.2**	159.6 + 9.4 5.50 + 0.14 34.5	n.d.	n.d.	n.d.
Isomaltose	K_m_ (mM) k_cat_ (s^−1^) k_cat_/K_m_ (M^−1^ s^−1^)	96.6 ± 1.9 0.702 ± 0.064 7.22	219 ± 16 0.404 ± 0.010 1.85	n.d.	n.d.	n.d.	n.d.
Lactose	K_m_ (mM) k_cat_ (s^−1^) k_cat_/K_m_ (M^−1^ s^−1^)	n.d.	n.d.	204 ± 45 2.91 ± 0.34 14.5	n.d.	n.d.	n.d.
Laminaribiose	K_m_ (mM) k_cat_ (s^−1^) k_cat_/K_m_ (M^−1^ s^−1^)	n.d.	n.d.	143 ± 11.6 4.69± 0.16 32.9	>250 ± 20 ^b^ >1.03 ± 0.21 ^b^ 3.96 ^b^	n.d.	n.d.
Gentiobiose	K_m_ (mM) k_cat_ (s^−1^) k_cat_/K_m_ (M^−1^ s^−1^)	132.1 ± 11.7 2.88 ± 0.12 21.9	n.d.	**89.8 ± 7.5** **36.5 ± 0.6** **408**	**51.6 ± 3.8** **6.29 ± 0.32** **122.3**	n.d.	n.d.

^a^ data from [10], determined at pH 5.5 and the ferrocenium ion at a fixed concentration. ^b^ maximum velocity (v_max_) could not be reached due to limited solubility of substrate. n.d., not determined.

## Data Availability

The data presented in this study are available within the article and the supplementary material.

## Supplementary Materials

The following are available online at https://www.mdpi.com/article/10.3390/jof7100873/s1, Figure S1: Western Blot analysis of His-tagged proteins in the culture supernatants of *K. phaffii* expression hosts (A) and SDS-polyacrylamide gel electrophoresis of purified AA3_2 glucose oxidoreductases (B). GOx II from *Aureobasidium subglaciale* (1), GDH II from *Trichoderma virens* (2), GDH III from *Rhizoctonia solani* (3), and GDH III from *Ustilago maydis* (4). The glycosidase PNGase F is indicated by the arrow, M indicates the molecular mass markers; Figure S2: Molecular mass determination by SEC-MALS. (a) GOx II from *Aureobasidium subglaciale*, (b) GDH II from *Trichoderma virens*, (c) GDH III from *Rhizoctonia solani*, and (d) GDH III from *Ustilago maydis*. The absorbance measured is shown by orange lines (280 nm) and blue lines (450 nm); Figure S3: Absorption spectra of glycosylated GOx/GDHs (a) GOx II from *Aureobasidium subglaciale*, (b) GDH II from *Trichoderma virens*, (c) GDH III from *Rhizoctonia solani*, and (d) GDH III from *Ustilago maydis*. The spectra of the oxidized enzymes are shown as solid lines, and the spectra of GOx/GDHs reduced by adding 100 mM glucose are shown as dashed lines; Figure S4: Effect of temperatures to enzyme activity determined by thermal inactivation assays (A), and thermal stability curves plotted against fluorescence signal determined by the ThermoFAD assay (B); Table S1: Analysis of gene and protein sequences for selected glucose oxidoreductases; Table S2: Sequence similarity matrix of newly studied representatives of the GOx/GDH family and sequences of AA3_2 glucose oxidoreductases with known crystal structures.
