# Supplementary material for: Characterization of Fungal FAD-Dependent AA3_2 Glucose Oxidoreductases from Hitherto Unexplored Phylogenetic Clades
Source: J Fungi (Basel). 2021 Oct 17;7(10):873. doi: 10.3390/jof7100873 (PMC8537048; doi:10.3390/jof7100873)
Supplement: Supplementary file 1 [file jof-07-00873-s001.zip › jof-1380901-supplementary.pdf]

Supplementary material

Characterization of fungal FAD-dependent AA3_2 glucose oxidoreductases from hitherto unexplored phylogenetic clades

Sudarma Dita Wijayanti [1,2], Leander Sützl [1], Adèle Duval [1], and Dietmar Haltrich[1,*]

[1] Laboratory of Food Biotechnology, Department of Food Science and Technology, BOKU - University of Natural Resources and Life Sciences Vienna, Muthgasse 11, A-1190 Wien, Austria; sudarma.wijayanti@boku.ac.at (S.D.W.); leander.suetzl@boku.ac.at (L.S.), adele.duval@savencia.com (A.D.)

[2] Department of Agricultural Product Technology, Brawijaya University, Veteran, 65145 Malang, East Java, Indonesia

* Correspondence: dietmar.haltrich@boku.ac.at (D.H.)

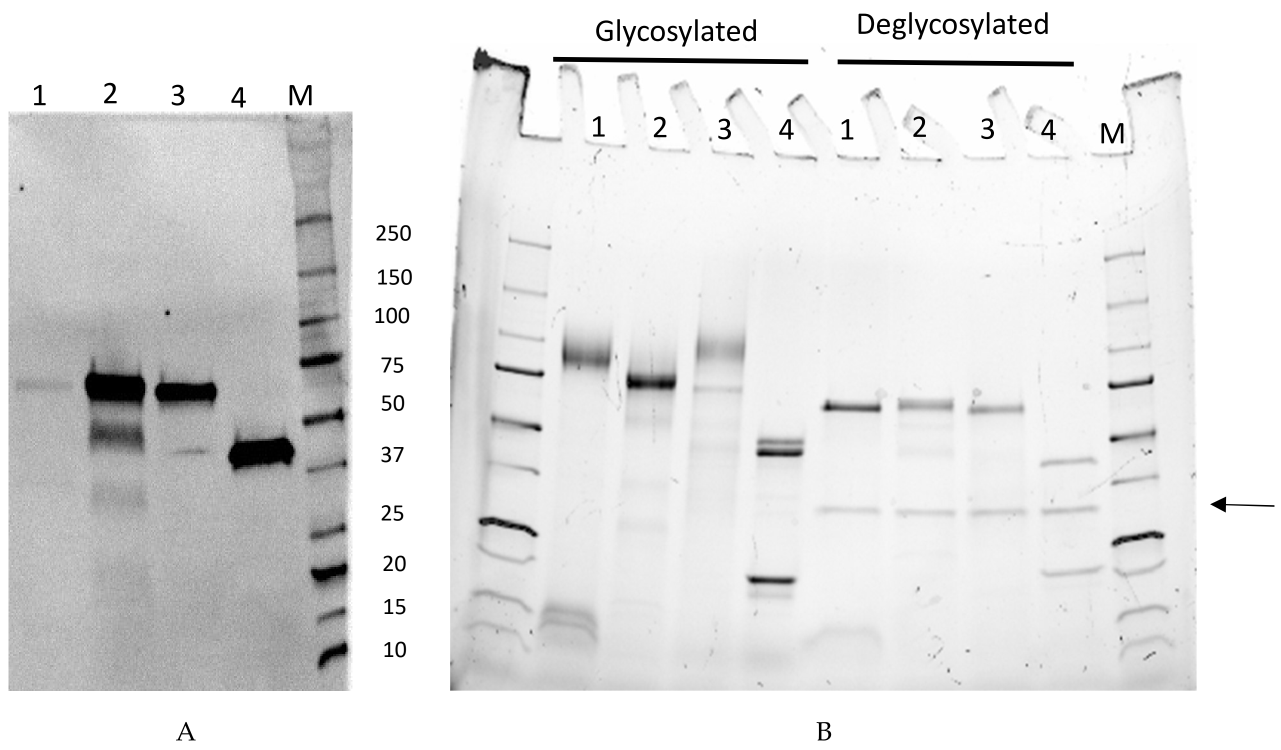

Figure S1. Western Blot analysis of His-tagged proteins in the culture supernatants of *K. phaffii* expression hosts (A) and SDS-polyacrylamide gel electrophoresis of purified AA3_2 glucose oxidoreductases (B). GOx II from *Aureobasidium subglaciale* (1), GDH II from *Trichoderma virens* (2), GDH III from *Rhizoctonia solani* (3), and GDH III from *Ustilago maydis* (4). The position of PNGase F is indicated by an arrow, M indicates the molecular mass markers.

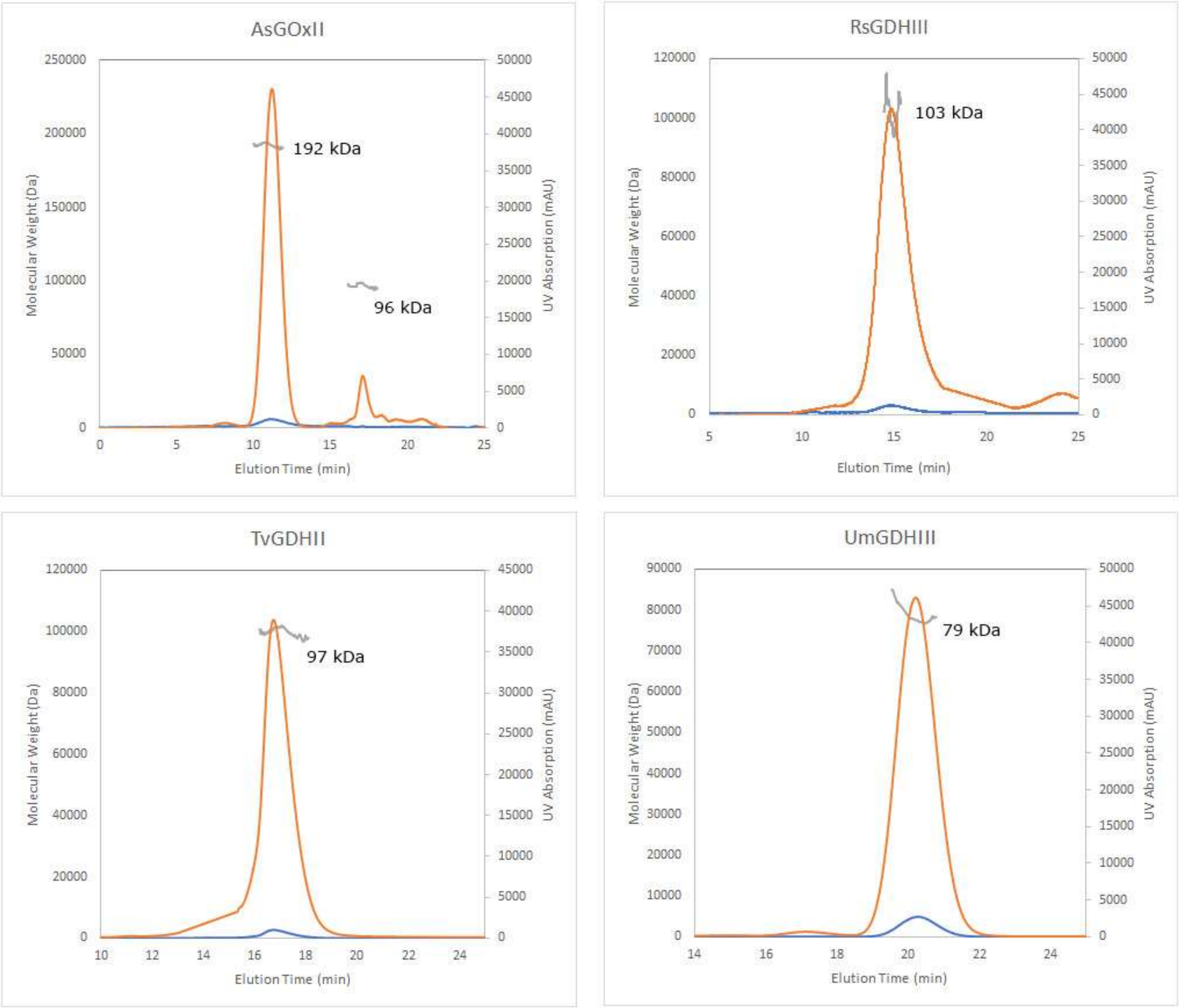

Figure S2. Molecular mass determination of AA3_2 glucose oxidoreductases by SEC-MALS. (a) GOx II from *Aureobasidium subglaciale,* (b) GDH II from *Trichoderma virens,* (c) GDH III from *Rhizoctonia solani,* and (d) GDH III from *Ustilago maydis*. The absorbance measured is shown by orange lines (280 nm) and blue lines (450 nm).

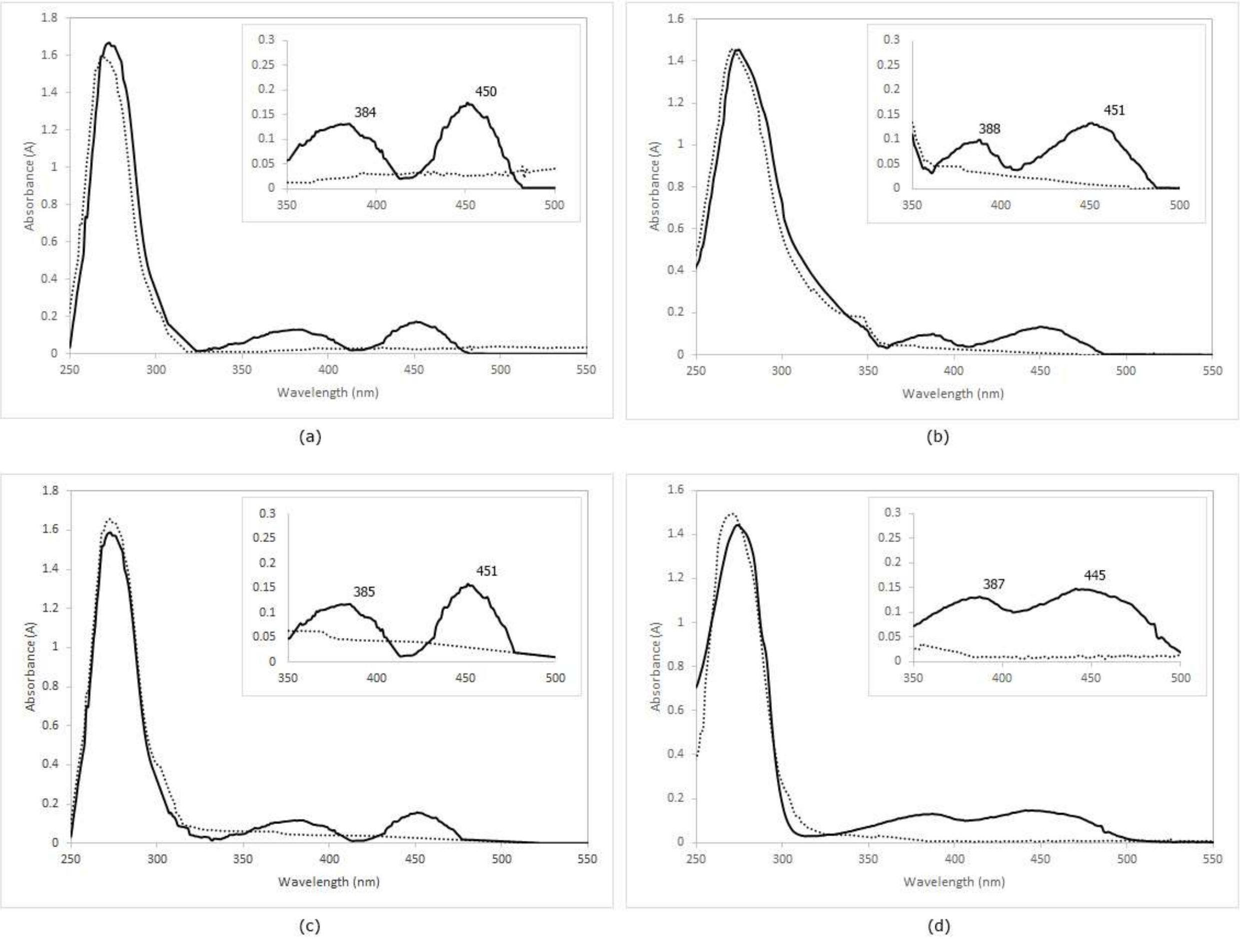

Figure S3. Absorption spectra of glycosylated GOx/GDH samples (a) GOx II from *Aureobasidium subglaciale*, (b) GDH II from *Trichoderma virens,* (c) GDH III from *Rhizoctonia solani,* and (d) GDH III from *Ustilago maydis*. The spectra of the oxidised enzymes are shown as solid lines, and the spectra of glucose oxidoreductases reduced by the addition of 100 mM glucose are shown as dashed lines.

A

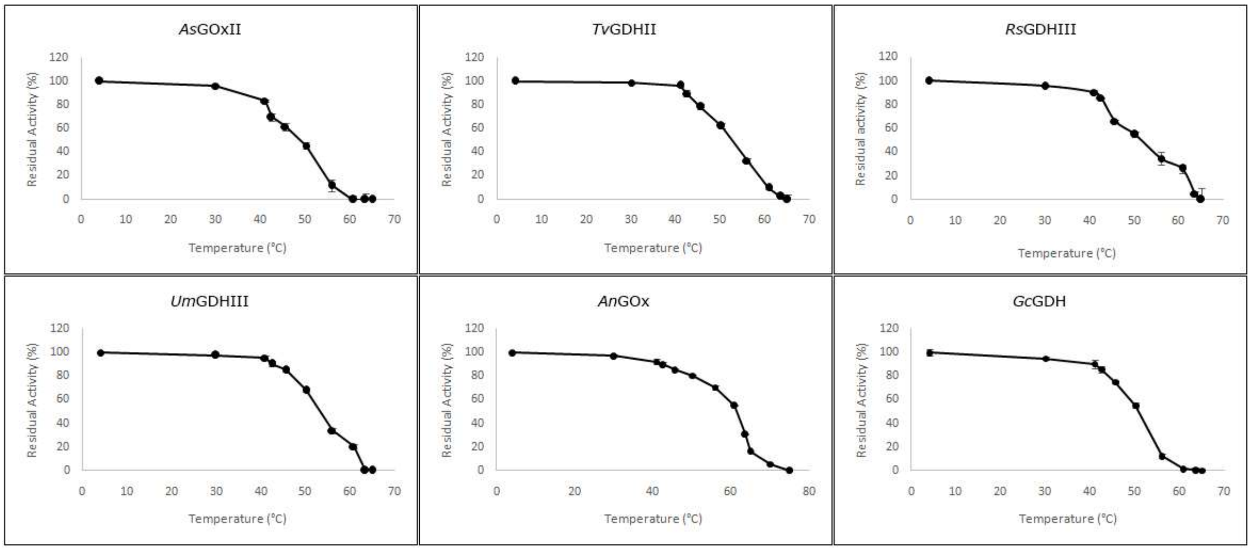

B

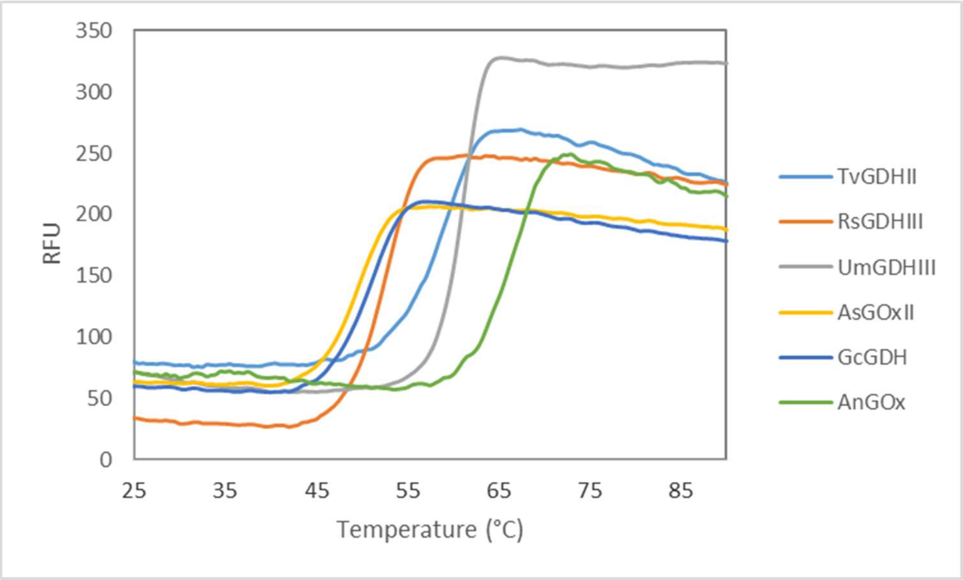

Figure S4. Effect of temperatures to enzyme activity determined by thermal inactivation assays (A), and thermal stability curves plotted against fluorescence signal determined by the ThermoFAD assay (B).

Table S1. Analysis of gene and protein sequences for selected glucose oxidoreductases

			Full length gene			Mature protein (as expressed)		
	**Enzyme name, source organism**	**GenBank entry**	**ORF (base pairs/amino acids)[a]**	**GC Content (%)[a]**	**Putative Signal Peptidase Cleavage Site[b]**	**Putative N Glycosylation Sites[c]**	**Calculated MW (Da)[d]**	**Calculated pI[d]**
1	*As*GOxII, *Aureobasidium subglaciale*	KEQ90431	1893/631	49	Cleavage site between position 16 and 17, probability: 0.8348	N7, N36, N73, N83, N184, N220, N368, N396	66187.36	5.18
2	*Tv*GDHII, *Trichoderma virens*	EHK19553	1842/ 614	48	Cleavage site between position 24 and 25, probability: 0.5057	N167, N227, N232, N291, N340, N492	64142.84	4.87
3	*Rs*GDHIII, *Rhizoctonia solani*	CEL62789	1875/625	49	Cleavage site between position 19 and 20, probability: 0.7147	N12, N189, N371, N490	64909.80	7.81
4	*Um*GDHIII, *Ustilago maydis*	KIS68464	1974/658	49	Cleavage site between position 23 and 24, probability: 0.8527	N255, N380	67633.63	9.37
	*An*GOx, *Aspergillus niger*	CAA34197	1887/629	49	Cleavage site between position 16 and 17, probability: 0.4373	N49, N95, N167, N174, N264, N394, N479	64876.18	5.26
	*Gc*GDH, *Glomerella cingulata*	AER13600	1818/606	61	Cleavage site between position 16 and 17, probability: 0.5703	N55, N84, N176, N184, N233, N255, N339, N389	62997.89	4.79

[a] SnapGene Viewer
[b] identified with SignalP 5.0
[c] potential N-glycosylation sites identified with the program NetNGlyc 1.0
[d] Compute pI/Mw (Expasy Proteomics)

Table S2. *Sequence similarity matrix of newly studied representatives of the GOx/GDH family and sequences of AA3_2 glucose oxidoreductases with known crystal structures.* Sequence similarity values are given in %. Values > 40% are highlighted in bold.

*Pc*GDHIII, GDH III from *Pycnoporus cinnabarinus*; *An*GOx, GOx from *Aspergillus niger*; *Af*GDHI, GDH I from *Aspergillus flavus*; *As*GOxII, GOx II from *Aureobasidium subglaciale*; *Tv*GDHII, GDH II from *Trichoderma virens*; *Rs*GDHIII, GDH III from *Rhizoctonia solani*; and *Um*GDHIII, GDH III from *Ustilago maydis.*

	*Pc*GDHIII (*Pc*ODH)	*An*GOx	*Af*GDHI	*As*GOxII	*Tv*GDHII	*Rs*GDHIII	*Um*GDHIII
*Pc*GDHIII (6XUT)	100	36.07	37.74	37.14	34.43	**59.73**	**45.92**
*An*GOx (1CF3)	-	100	34.78	32.41	31.42	34.54	32.13
*Af*GDHI (4YNT)	-	-	100	35.15	**42.20**	36.68	38.41
